# A widely used pan-isoform-FBXW7 antibody used in cell cycle studies does not detect FBXW7

**DOI:** 10.1080/15384101.2023.2210044

**Published:** 2023-05-14

**Authors:** Hannah Ennis, Denise M McDonald

**Affiliations:** Wellcome-Wolfson Institute for Experimental Medicine, Queen’s University Belfast, Belfast, UK

**Keywords:** Endothelial cells, tumour suppressor gene, protein degradation, western blot, FBXW7

## Abstract

FBXW7 is the substrate recognition component of the E3 ubiquitin ligase SCF^FBW7^ complex which controls the levels of CYCLINE, c-MYC and HIF1α proteins crucial for cell growth and differentiation. Mutations in FBXW7 are frequently associated with tumourigenesis. While examining FBXW7 regulation we were compelled to reevaluate a commonly used anti-FBXW7 antibody. Retinal microvascular endothelial cells (RMEC) were exposed to normoxia (21% oxygen) or hypoxia (1% oxygen) for 24 h or treated with MG132 and protein extracted for western blotting. Flag-tagged FBXW7-α, β or γ isoforms were transfected into HEK293A cells and processed using denaturing and native extraction protocols for western blotting or immunoprecipitation analysis. Two anti-FBXW7 antibodies were used, one raised to the unique FBXW7α N-terminus and the other to the C-terminus region common to all isoforms. Initial studies showed that the pan-isoform C-terminus antibody detected a single 64kDa band in RMEC rather than any of the predicted sizes for FBXW7. In contrast, expression of the isoform-specific constructs, detected with an anti-Flag antibody, confirmed the expected migratory distance of 110kDa, 68kDa and 65kDa for α, β and γ respectfully. Similarly, the N-terminus FBXW7α antibody also detected the 110kDa product. Notably, the C-terminus antibody did not recognize any of the isoforms but continued to detect a 64kDa band in all samples, including the non-transfected controls. Immunoprecipitation confirmed this lack of specificity and the inability to detect overexpressed or endogenous FBXW7α in HEK293A cells and RMEC. A commonly used C-terminus FBXW7 antibody does not detect FBXW7 under standard western blotting conditions.

## Introduction

FBXW7 is the substrate recognition component of the E3 ubiquitin ligase SCF^FBW7^ (a complex also consisting of SKP1 and CULLIN1) which acts as an important signaling hub controlling the levels of a wide range of proteins important for cell growth and differentiation including c-MYC, CYCLINE, NOTCH, KLF2 and SREBP (sterol regulatory binding protein) [1–3]. FBXW7 is produced in one of three isoforms which differ in their sub cellular localization to the nucleus (α isoform), the cytoplasm (β isoform) and the nucleolus (γ isoform) [4]. FBXW7 is frequently absent or mutated in cancer causing the upregulation of a range of tumour promoting oncogenes such as c-MYC [5]. Accordingly, FBXW7 depleted cells are resistant to death and many studies have linked it to a range of cancers. In addition, a recently discovered germline mutation in FBXW7 was shown to be associated with impaired protein turnover in a neurodevelopment disorder [6]. Therefore, studies describing how FBXW7 is regulated are crucial for the development of anti-cancer therapies and future understanding of disorders that occur during development.

Structurally, all three FBXW7 isoforms are homodimers that contain a variable N-terminus region (encoded by exon 1), a common D domain, an F box moiety and a series of WD repeat motifs close to the C-terminus of the protein. FBXW7 acts as a recruitment platform bringing together substrate and ubiquitin modifying enzymes. Specifically, substrate binding occurs in the WD repeat domain bringing it close to SKP1 and CULLIN1 bound to the adjacent F box domain. These linker proteins facilitate the addition of ubiquitin to the substrate which is targeted for proteasomal degradation or further processing. Crucially, therefore, substrate binding and turnover are dependent on the abundance of FBXW7, also tightly controlled by self-ubiquitination and proteasomal degradation [2]. Accordingly, tumour progression is associated with decreased FBXW7 stability and an overabundance of tumour promoting substrates. Conversely, factors that stabilize FBXW7, as occurs in the presence of the deubiquitinating enzymes USP9X and USP28, are tumour suppressive [7,8]. Therefore, considering the importance of FBXW7 in controlling cell fate decisions, a better understanding of how it is regulated is essential to understanding its role in health and disease.

In addition to its tumour suppressor function, FBXW7 also facilitates development of the cardiovascular system, a feature most notably displayed in FBXW7 knockout animals which present with major vascular impairment due, in part, to unrestrained NOTCH activation [9]. Interestingly, however, further follow-on studies using EC-specific knockout models indicate a context dependent effect with evidence that FBXW7 can both positively and negatively regulate angiogenesis depending on the tissue or organ examined [10,11]. Whether these differences are mediated by cell specific expression of particular substrates or to variability in FBXW7 regulation remains to be determined. A major driver of developmental angiogenesis is tissue hypoxia acting through the hypoxia sensor HIF1α. FBXW7 has been shown to be involved in controlling HIF1α levels and also HIF1α effector proteins such as c-MYC [12–14]. How these opposing functions are regulated are also poorly understood, indicating a need for further study of FBXW7 regulation in vascular endothelial cells.

Defining the role of FBXW7 and its associated isoforms is dependent on the availability of well characterized research tools. Antibodies, are one such tool that have played a crucial role in defining the role of proteins of interest in disease. Unfortunately, however, through our own studies investigating FBXW7 in vascular biology, we have found that a widely used antibody is unreliable. Here we provide evidence of its inadequacies. Moreover, investigating FBXW7 levels is complicated by its rapid turnover by ubiquitin modification and proteasomal degradation. In addition, the dominant α isoform runs at an apparent molecular weight different from its actual size [1]. Taking this into consideration, here we compared two commonly used antibodies to FBXW7, one directed to the C-terminus (residues 650-C-terminus) end of FBXW7, a region common to all three isoforms and one that targets the N-terminus of the protein that is unique to the α isoform for their effectiveness to detect FBXW7. We found that the C-terminus antibody does not detect FBXW7 under standard conditions used in western blotting protocols. By comparing the position of the epitopes noted in the 3D structure of FBXW7 it is likely that the C-terminus epitope forms part of a β-sheet tertiary structure and upon denaturing is not effectively detected by antibodies that were raised to this domain [15,16]. We would therefore urge caution in using this antibody to determine FBXW7 protein levels in western blotting.

## Experimental procedures

### Primary retinal microvascular endothelial cell (RMEC) isolation and culture

Primary retinal microvascular endothelial cells (RMEC) were isolated and a homogenous population confirmed by immunofluorescence as described previously [17]. Briefly, retinas were extracted from bovine eyes obtained from the local abattoir and stored on ice. Retinas were gently washed in isolation media (minimum essential medium (MEM), Thermofisher) containing 30m M HEPES, 2.5 mM Glutamine (Thermofisher) and Primocin (Invitrogen, 126μg/ml) and cut into fragments with sterile forceps and a scalpel. The media containing fragments was then transferred to a homogenizer and homogenized eight times with a harsh, circular stroke. The homogenate was centrifuged at 1,600 RPM for 10 minutes at 4°C. The supernatant was removed and the pellet re-suspended in 5 ml isolation media prior to filtering through a 85 μM gauze. The tissue remnants collected on top of the gauze were then placed in an enzyme cocktail (10 ml) containing Pronase (5 mg, Sigma), DNase (6 mg, Worthington Biochemicals) and Collagenase (3 mg, Sigma) for 27 minutes at 37°C in a 5% CO_2_ incubator. To stop the reaction, 10mls ice cold isolation media was added and the resultant enzyme digest was filtered through a 53 μM gauze. The blood vessel fragments remaining on the gauze were placed in 10mls isolation media and centrifuged at 1,100 RPM for 10 minutes at 4°C. The supernatant was removed and the pellet re-suspended in 5mls of RMEC growth media, Dulbecco’s Modified Eagle Medium (DMEM) supplemented with 20% porcine serum, heparin (5μg/ml) and insulin (0.38μg/ml). The re-suspended retinal vessels were plated onto gelatin coated T25 flasks. RMEC were sub-cultured in DMEM containing 20% porcine serum (Thermo Fisher, 26250084), heparin (VWR, A3004) and insulin (Thermo Fisher, RP-10908) until passage two at which point porcine serum was reduced to 10%. All primary cells and cell lines were tested routinely for the absence of mycoplasma. For hypoxia treatment, RMECs were seeded on gelatin-coated 6-well plates at 2.5 × 10^5^ cells per well the day prior to treatment. Spent media was removed and replaced with 1 ml fresh pre-warmed RMEC growth media prior to placement in 24h normoxia (21% O_2_, 5% CO_2_) or hypoxia (1% O_2_, 5% CO_2_) using a InvivO_2_ 400 hypoxia workstation (Baker Ruskinn). Cells were collected after 24 h and processed for western blotting. For MG132 treatment, MG132 (Sigma, M7449) was added to RMECs at a final concentration of 20 µM as described previously [5,8]. Vehicle control, dimethyl sulfoxide (DMSO) (Sigma, 276855), was added at a final concentration of 0.2%. Treatments were added directly to the media bathing the cells and incubated for 6 h at 37°C in a 5% CO_2_ incubator. FBXW7 protein levels were analyzed by western blotting as described below.

### Western blotting

#### Sample lysis and SDS-PAGE electrophoresis

Tissue culture plates containing cells were placed on ice and cells washed with ice-cold phosphate buffered saline (PBS). For samples collected using IGEPAL lysis buffer, 200 µl of ice-cold IGEPAL buffer (1% Deoxycholate, 100 mM 1 M Tris-HCl pH7.4, 0.1% SDS, 0.01% IGEPAL, plus EDTA-free protease inhibitor cocktail, Roche) was added and cells scraped using a cell scraper and collected into Eppendorfs. Samples were placed on a spinning wheel at 4°C for 30 minutes then centrifuged at 13,000 RPM for 10 minutes at 4°C. After centrifugation, supernatant was removed and NuPage lithium dodecyl sulfate (LDS) sample buffer (4x), ThermoFisher, NP0008;-containing NuPage sample reducing agent (10×) added to each sample to yeild a final buffer concentration of x1. Samples were boiled at 95°C for 5 minutes prior to placing on ice. To facilitate fast lysis under denaturing conditions, protein samples were collected in 200 µl of ice-cold loading buffer (NuPage LDS sample buffer (1x), – Lithium dodecyl sulfate (pH 8.5), SERVA Blue G250 and phenol red; NuPage sample reducing agent, ThermoFisher, NP0004 (50 mM dithiothreitol (DTT final concentration)) was added per well of a 6-well plate. Lysed cells were removed using a cell scraper and collected into Eppendorfs and samples sonicated on ice at 4 amps for 30 seconds. Samples were boiled at 95°C for 5 minutes and returned to ice. Denaturing SDS-PAGE gels were made using the Bio-Rad casting system with 4% acrylamide for the stacking gel and 8% for the resolving gel.

### Immunoblotting parameters

After electrophoresis, proteins were transferred to Polyvinylidene fluoride (PVDF) membrane (Immobilon-P PVDF, Merck, IPVH00010). PVDF membrane was pre-soaked in methanol for 5 minutes prior to soaking in transfer buffer containing 20% (v/v) methanol. Blotting paper (GE Healthcare, Analab, 3030672) was also pre-soaked in transfer buffer. Gels were then transferred to the membrane at 150 mA for 16 h at 4°C.

### Western blot analysis of protein samples

After transfer, nonspecific binding of antibody to the membranes was prevented by incubation with blocking buffer consisting of 5% (w/v) skimmed milk powder, PBS and 0.1% Tween-20 (PBS-Tween) on a platform rocker for 1 h at room temperature (RT). Membranes were then incubated with primary antibody, anti-FBXW7 directed against the C-terminus raised using a synthetic peptide corresponding to amino acids 650 to the C terminus of FBXW7, rabbit polyclonal, Abcam, ab109617; 1:1000, anti-FBXW7 directed against the N-terminus residues 1–50 of human FBXW7α, rabbit polyclonal, Bethyl Laboratories, A301-720A; 1:4000; anti-Flag, mouse monoclonal, Sigma, F3165; 1:10000, anti-β-ACTIN, mouse monoclonal, Sigma, A5316; 1:5000 or anti-HIF1α, mouse monoclonal, BD Biosciences, 610958; 1:1000 also diluted in blocking buffer on a platform rocker for 1 h at RT. Following incubation, membranes were washed in PBS-Tween three times in 20 minute intervals. Membranes were then incubated with horseradish peroxidase (HRP)-conjugated secondary antibody goat anti-rabbit HRP, Cell Signaling, 7074S; 1:2000 or goat anti-mouse HRP, Cell Signaling, 7076S; 1:2000 on a platform rocker for 1 h at RT. Membranes were then washed in PBS-Tween as before. To develop the blots, Immobilon western chemiluminescent reagent was used (Millipore, WBKLS0500). Images were developed using the G: Box (Syngene) and GeneSys software (Syngene). Densitometry was determined using images with no saturation of western blot bands and measured using NIH ImageJ software. β-ACTIN was routinely used to correct for equal loading of protein.

### Transduction of HEK 293A cells with FBXW7 isoform plasmids

Isoform specific constructs (cDNA clone expression plasmids corresponding to α, β and γ FBXW7) were purchased from Sino Biological all containing N-terminus Flag epitope tags. Plasmids were prepared using endotoxin free plasmid isolation kits (Qiagen, 12362). Human embryonic kidney (HEK) 293A were routinely cultured in DMEM supplemented with 10% Fetal calf serum (FCS) and seeded at 2.5 × 10^5^ cells per well in 6-well plates the day prior to transfection. HEK 293A cells were transfected with 1 µg plasmid DNA using Turbofect (ThermoFisher, R0532) transfection reagent in Opti-MEM I Reduced Serum Medium (ThermoFisher, 31985062) and incubated at 37°C (5% CO_2_) for 48 h post-transfection before collection of lysate for western blotting.

### Immunoprecipitation of FBXW7α by anti-Flag and anti-N-terminus FBXW7 antibodies

HEK 293A cells were transfected with FBXW7α or GFP as control for 48 h as described above and FBXW7 immunoprecipitated with an anti-Flag, anti-C terminus FBXW7, anti-N-terminus FBXW7 or control IgG antibodies using IgG agarose beads. Briefly, HEK 293A cells were plated on T75 flasks the day prior to transfection. Cells were transfected with 7.8 µg of GFP or FBXW7α plasmid DNA (per T75) combined with Turbofect (ThermoFisher, R0532) transfection reagent in Opti-MEM I Reduced Serum Medium (ThermoFisher, 31985062). HEK 293A protein samples were collected 48 h post-transfection and immunoprecipitation carried out. On ice, T75 flasks were washed with 5 ml ice-cold PBS. Ice-cold 1× CST lysis buffer (1 ml; Cell Signaling, 9803) supplemented with 1mM PMSF (Merck, 93482), 5mM Sodium fluoride (NaF, Merck, 67414) and EDTA-free protease inhibitor cocktail (Roche/Sigma, cOmplete^TM^) was added, and cells incubated for a further for 15 minutes on ice. Cell lysates were harvested by scraping and collected into an Eppendorf. Lysate was clarified by centrifugation at 13,000 RPM for 15 minutes at 4°C and supernatant was removed into a new Eppendorf. BCA was carried out to determine protein amount and 1 mg of protein sample was incubated with 2 µg of each antibody overnight on a rotating wheel at 4ºC. An aliquot of total lysate was removed and NuPage LDS sample loading buffer containing NuPage sample reducing agent added and the sample boiled at 95°C for 5 minutes and stored at −20°C. The following morning, 20 µl A/G agarose beads (Insight Biotechnology, SC-2003) were added to each protein-antibody mix and the samples placed on a rotating wheel for 2 h at 4°C. After incubation, samples were centrifuged at 2,500 RPM for 1 minute at 4°C to separate the protein-antibody-bead fraction from the unbound fraction which was removed. The remaining pellet was washed by adding 800 µl ice-cold 1× CST cell lysis buffer (containing freshly added protease inhibitors), vortexing briefly, placing on a rotating wheel for 10 minutes at 4°C and re-centrifuging; these steps were repeated 5 times. After washing, protein was eluated from the bead pellet by the addition of 100 µl neat (4×) NuPage LDS sample loading buffer containing reducing agent. Samples were boiled at 95°C for 5 minutes, placed on ice for 5 minutes, vortexed briefly and then centrifuged at 13,000 RPM for 5 minutes at 4°C. Immunoprecipitated protein samples were separated by SDS-PAGE in three individual blots and each one immunoblotted with anti-Flag, anti-C terminus or anti-N-terminus FBXW7 antibody as described above.

### Immunoprecipitation of endogenous FBXW7α in RMEC and 293A cells

RMEC and HEK 293A were plated on T75 flasks and incubated overnight at 37°C. The following morning, the flasks were placed on ice and washed twice with ice-cold PBS. After washing, 5 ml of ice-cold PBS was added, the cells were harvested by scraping and collected into falcons and the contents of two flasks per cell type pooled. Cells were collected by centrifugation at 1,500 RPM for 5 minutes at 4°C. The resulting cell pellets were re-suspended in 400 µl ice-cold 1× CST lysis buffer (Cell Signaling, 9803) supplemented with 1 mM PMSF, 5 mM NaF and a protease inhibitor tablet and incubated for 10 minutes on rotating wheel at 4°C. Lysate was clarified by centrifugation at 13, 000 RPM for 10 minutes at 4°C. BCA was carried out to determine protein amount. An aliquot of the lysate was removed for input sample comparison and 1× NuPage LDS sample loading buffer containing 1× NuPage sample reducing agent used to denature the protein. Samples were boiled at 95°C for 5 minutes and stored at −20°C. Lysate samples were pre-cleared with Dynabeads (ThermoFisher, 10004D) for 45 minutes on a rotating wheel at 4°C. After pre-clearing, lysate was removed from the Dynabeads using a magnetic rack and the lysate incubated with 1 µg antibody overnight on a rotating wheel at 4°C. The following morning, the antibody containing lysates were added to 50 µl Dynabeads and placed on rotating wheel for 1 hour at 4°C. After incubation, samples were placed on a magnetic rack to separate the beads containing the protein-antibody complexes from the remaining supernatant containing the unbound fraction. The bead pellets were then washed by adding 500 µl ice-cold 1× CST cell lysis buffer (supplemented with fresh protease inhibitors), mixed briefly, placed on a rotating wheel for 5 minutes at 4°C and the wash removed using a magnetic rack; these steps were repeated 3 times. After washing, protein was eluted from the beads by the addition of 60 µl neat (4×) NuPage LDS buffer containing reducing agent and vortexed briefly. Samples were boiled at 95°C for 5 minutes and placed on ice for 5 minutes prior to placing on a magnetic rack and the eluate removed from the beads into new Eppendorf’s. Protein samples were separated by denaturing gel electrophoresis as described above and transferred to PVDF membrane. Following transfer, protein samples, run on parallel gels, were detected using anti-FBXW7 N-terminus or anti-FBXW7 C-terminus antibodies and detected as described above.

## Results

### FBXW7 detection in western blots and effect of MG132 on FBXW7 immunoreactivity

**Figure 1.**
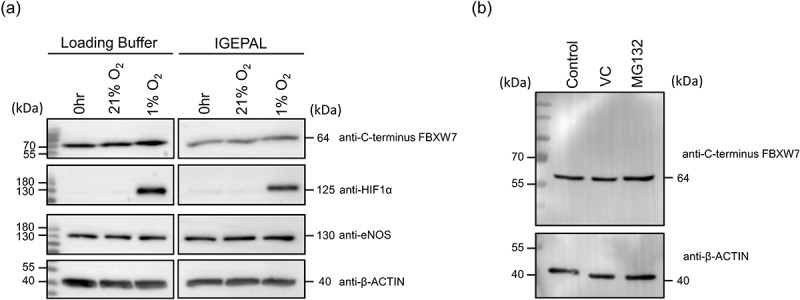
Detection of FBXW7 in western blot analysis after exposure of RMEC to hypoxia (a) or MG132 (b). (a) Cells were placed in normoxic (21% oxygen) or hypoxic (1% oxygen) conditions for 24h, collected in loading or IGEPAL buffer, separated by SDS-PAGE and processed by western blotting. An anti-C-terminus FBXW7 antibody detected a band of 64kDa in all samples. An anti-HIF1α antibody confirmed stabilization of HIF under hypoxia and anti-eNOS and anti-β-ACTIN antibodies confirmed equivalence of protein loading. (b) Effect of MG132 on the levels of the 64kDa band detected by the C-terminus FBXW7 antibody. RMEC were treated with MG132 or control for 6h before collection in loading buffer. Transferred protein was probed with an anti-C-terminus FBXW7 antibody. Equivalent amounts of protein were confirmed with an anti-β-ACTIN antibody.

RMEC exposed to normoxia (21% oxygen) or hypoxia (1% oxygen) were lysed directly in one of two buffers in preparation for western blotting. The first buffer (IGEPAL) contained the detergents IGEPAL and deoxycholate and is routinely used for studies requiring downstream processing such as radioimmunoassay or immunoprecipitation which require preservation of the protein tertiary structure. The second buffer (loading buffer) contained high levels of LDS to facilitate immediate denaturation of proteins. The latter extraction buffer is not compatible with the BCA reagent therefore differences in loading amounts were routinely corrected by immuno-detection of β-ACTIN. Notably, however, differences in total protein were usually negligible since equivalent amounts of cells were routinely seeded per treatment. As expected HIF1α was stabilized in cells exposed to hypoxia as observed by a band that migrated at 125kDa and that was only present in the cells exposed to 1% O_2_ for 24 h (Figure 1a). eNOS and β-ACTIN confirmed that equivalent amounts of protein were loaded for each treatment. Surprisingly, the FBXW7 C-terminus antibody detected a band corresponding to a molecular weight of 64kDa which was unchanged by hypoxia exposure. This size is counter to the actual molecular weight of any of the FBXW7 isoforms. Moreover, FBXW7α is typically the most highly expressed isoform in all cells and has been shown to migrate at an apparent molecular weight of approximately 110kDa in SDS-PAGE therefore, it was surprising that the C-terminus directed antibody only detected a band of 64kDa [1]. The FBXW7 family of proteins are short lived and their levels controlled by auto-ubiquitination and turnover by the proteasome. Thus, since it was possible that FBXW7 was subject to degradation, cells were treated with the proteasome inhibitor MG132. Again, the 64kDa band detected by the anti-C-terminus FBXW7 antibody did not show any significant increase in protein abundance when MG132 was applied (Figure 1b). This suggested the possibility that this particular band was nonspecific and not FBXW7.

### Use of isoform specific Flag-tagged constructs to determine detection by an anti-C-terminus FBXW7 antibody

**Figure 2.**
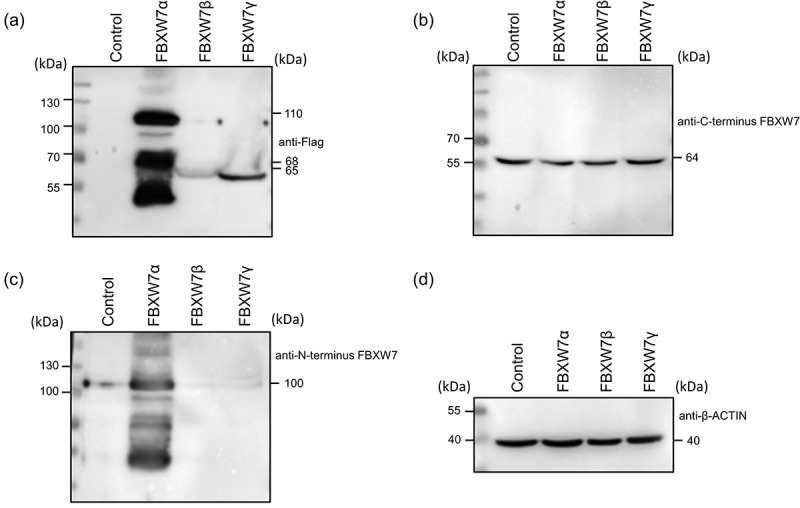
Comparison of antibody detection of α, β and γ FBXW7-Flag isoforms and differential detection of transfected FBXW7α-Flag by an anti-Flag and an anti-N-terminus but not an anti-FBXW7 C-terminus antibody. HEK 293A cells were transfected with α, β and γ FBXW7 isoforms for 48h and protein extracted in loading buffer. (a) The anti-Flagantibody shows the molecular weights of each of the three isoforms 79kDa predicted but 110kDa actual, 68kDa and 65kDa in α, β and γ transfected samples respectively but not in controls by an anti-Flag antibody. (b) Anti-C-terminus FBXW7 antibody shows a band at 64kDa in all samples even the untransfected controls. (c) Anti-N-terminus FBXW7 antibody detected a band at 110kDa in only the FBXW7α transfected samples. (d) β-ACTIN confirmed equivalency of protein loading.

Next, in order to determine if the C-terminus FBXW7 antibody could detect each of the individual FBXW7 isoforms, as would be expected from the position of the epitope to a region common to all three, each isoform was expressed in 293A cells. Importantly, each construct contained an N-terminus positioned Flag tag to facilitate detection with an anti-Flag antibody. Overexpression and comparison of all three FBXW7 isoforms yielded bands at the correct molecular weight for each isoform when probed with an anti-Flag antibody (Figure 2a). On denaturing SDS-PAGE the Flag antibody showed bands at 110kDa for α, 68kDa for β and 65kDa for γ as expected. The C-terminus antibody however only detected a band at 64kDa for all three isoforms which was present in all samples and, notably, not obviously different in intensity to control non-transfected samples (Figure 2b). In addition, a second commercially available anti-FBXW7 antibody that targets the N-terminus domain of FBXW7α and therefore is isoform specific, also detected a band of 110kDa exclusively in the cells transfected with FBXW7α and not with the β or γ isoforms. Together, this confirmed that the α isoform runs at an apparent molecular weight of 110kDa.

### Immunoprecipitation of FBXW7α with an anti-N-terminus and an anti-Flag antibody but not an anti-C-terminus antibody

**Figure 3.**
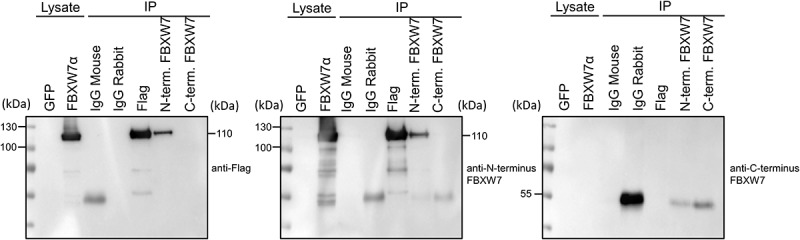
Immunoprecipitation of FBXW7α with anti-Flag, anti-N-terminus and C-terminus FBXW7 antibodies. HEK 293A cells were transfected with FBXW7α or GFP control plasmid. Following cell lysis protein was immunoprecipitated with anti-N-terminus FBXW7, anti-Flag, anti-C-terminus FBXW7 or control IgG antibody. Enriched proteins were separated on three separate gels and detected with anti-Flag, anti-N-terminus FBXW7 or anti-C-terminus FBXW7 antibodies.

Transfected cells were immunoprecipitated with anti-N terminus Fbxw7, anti-Flag, anti-C terminus FBXW7 or IgG control antibodies and the resulting enriched protein detected by each of these antibodies in parallel blots (Figure 3; three independent experiments were conducted and representative blots from one are shown). The anti-N terminus antibody used for enrichment showed a band at 110kDa in the FBXW7α transduced samples that was not present in the GFP transfected control lysates or the IgG mouse or IgG rabbit controls. The bands at 50kDa correspond to the heavy chain present in IgG rabbit antibody lanes. Detection using the anti-Flag showed a similar pattern to that found for the anti-N-terminus antibody. Similarly, the anti-Flag antibody also immunoprecipitated FBXW7α. In contrast, for the anti-C-terminus antibody, no band was present at 110kDa in GFP or FBXW7α transfected samples.

### Detection of endogenous FBXW7α in RMEC and effect of hypoxia and MG132

**Figure 4.**
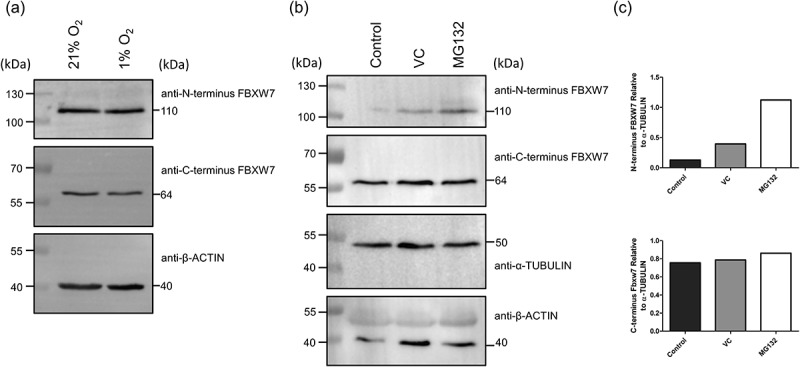
Detection of endogenous FBXW7α with an anti-N-terminus but not an anti-C-terminus FBXW7 antibody in RMEC exposed to hypoxia (a) or treated with MG132 (b). (a) RMEC were placed in normoxic (21% oxygen) or hypoxic (1% oxygen) conditions for 24h or (b) treated with MG132 or control for 6h before collection in loading buffer. Protein was separated by SDS-PAGE electrophoresis and transferred to PVDF membrane which was probed with an anti-N-terminus or an anti-C-terminus FBXW7 antibody. Equivalent amounts of protein were confirmed with an anti-β-ACTIN and anti-α-TUBULIN antibody. (c) Bands were quantified by densitometry and expressed relative to α-TUBULIN. the anti-C-terminus FBXW7 antibody detected a band of 64kDa in all samples whereas the anti-N-terminus detected a band at 110kDa corresponding to FBXW7α.

The ability of the anti-N-terminus antibody to detect endogenous FBXW7α was confirmed in RMEC subject to normoxia or hypoxia (Figure 4a) or treated with MG132 (Figure 4b). In contrast, as before, the anti-C-terminus antibody continued to detecta band at 64kDa. In addition, as opposed to the lack of any change in immunoreactivity of the 64kDa species in response to MG132 treatment there was an increase in the abundance of the 110kDa band corresponding to FBXW7α (Figure 4c).

### Comparison of anti-N terminus and anti-C terminus antibodies to immunoprecipitate endogenous FBXW7α

**Figure 5.**
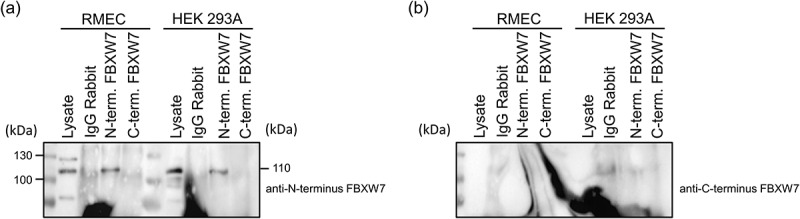
Immunoprecipitation of endogenous FBXW7α with an anti-Flag and anti-N-terminus but not an anti-C-terminus FBXW7 antibody in RMEC and HEK 293A cells. RMEC and HEK 293A cell protein samples were immunoprecipitated with anti-N-terminus, anti-C-terminus or control IgG antibody. Enriched proteins were separated on two independent gels and detected with (a) anti-N-terminus FBXW7 and (b) anti-C-terminus FBXW7 antibodies.

In agreement with the overexpression analysis, immunoprecipitation of endogenous FBXW7α in RMEC and HEK293A cells was demonstrated with the anti-N-terminus antibody, which yielded a product of 110kDa indicative of the α isoform (Figure 5a-b) but not the anti-C-terminus antibody. Together this indicates the efficacy of the anti-N-terminus but not the anti-C-terminus antibody to detect endogenous FBXW7α.

## Discussion

FBXW7 plays a pivotal role in controlling the levels of a broad range of cell fate determining proteins. Unsurprisingly, given its importance, almost 700 papers studying FBWX7 have been published in the last 5 years (Pub.Med.gov). A significant number of these publications, however, have used a commercially available antibody that, through our studies of FBXW7, we discovered fails to detect FBXW7. Specifically, through an original focus on FBXW7 regulation in the microvasculature we observed that this antibody does not reliably detect FBXW7 protein levels in western blotting protocols. This manuscript describes these studies in order to alert the scientific community of these defects and to urge caution in using this tool to investigate this important tumor suppressor gene.

Initially, using a variety of stimuli, such as exposure to hypoxia and treatment with MG132 we showed that an antibody directed to the C-terminus of FBXW7 detected a band on SDS-PAGE that migrated at 64kDa, a size that did not correspond to the actual or apparent molecular weights of any of the three FBXW7 isoforms; FBXW7α 110kDa, FBXW7β 68-70kDa and FBXW7γ 65-66kDa. As a protein, whose levels are controlled by proteasomal degradation, we also observed that the proteasome inhibitor MG132 had no effect on protein abundance. In addition, the expression pattern of the 64kDa band was unchanged by MG132. This finding, along with its unexpected migratory property, suggested a revaluation of the antibody was necessary and prompted us to review commonly used antibodies to FBXW7 for their effectiveness. In order to do this we used plasmids expressing cDNAs for each of the isoforms, all with N-terminal Flag tags. In addition, we also used a second FBXW7 antibody that is raised to an epitope in the N-terminus of the α isoform. Using these constructs, we showed that FBXW7β and FBXW7γ migrated at their predicted molecular weights and FBXW7α at its apparent molecular weight of 110kDa as evidenced by the banding pattern detected with an anti-Flag antibody [1]. Notably, consistently, the anti-C-terminus directed antibody continued to detect a single band at 64kDa that was present in all samples regardless of treatment. In contrast, the α-N-terminus antibody specifically detected the α isoform and not β or γ and in accordance with the anti-Flag antibody detected a band at 110kDa. The epitope that the anti-C-terminus antibody is raised against resides in the common C-terminus region of FBXW7 and it should therefore recognize and detect all three isoforms. Consequently, we tested the possibility that the anti-C-terminus antibody could only detect the protein in a more native conformation and not when fully denatured. To do this we used immunoprecipitation to compare the ability of each antibody, the anti-N-terminus, anti-C-terminus and anti-Flag to pull down FBXW7α. Notably again, only the N-terminus and the Flag antibodies were able to immunoprecipitate FBXW7α and not the C-terminus antibody.

Next, in order to compare the ability of each antibody to detect endogenous FBXW7 we again used primary endothelial cells subject to hypoxia and MG132. These findings again demonstrated that the FBXW7 N-terminus directed antibody could efficiently recognize endogenous FBXW7α whereas the C-terminus directed antibody did not. In this experiment, in contrast to the lack of change noted for the C-terminus-derived 64kDa band, MG132 treatment increased the abundance of the 110kDa species detected by the N-terminus antibody. Interestingly, this effect of MG132 is less marked than that observed for overexpressed FBXW7, in line with other studies using MG132 or cycloheximide to measure endogenous FBXW7 stability or half-life [2,5,8]. Further evidence of the differential effectiveness of the N-terminus and not the C-terminus antibody to detect FBXW7 was shown by the ability of the former and not the latter to immunoprecipitate endogenous FBXW7 in RMEC and in HEK293A cells. Together, these findings provide a framework for future studies investigating FBXW7 function in endothelial cells.

**Figure 6.**
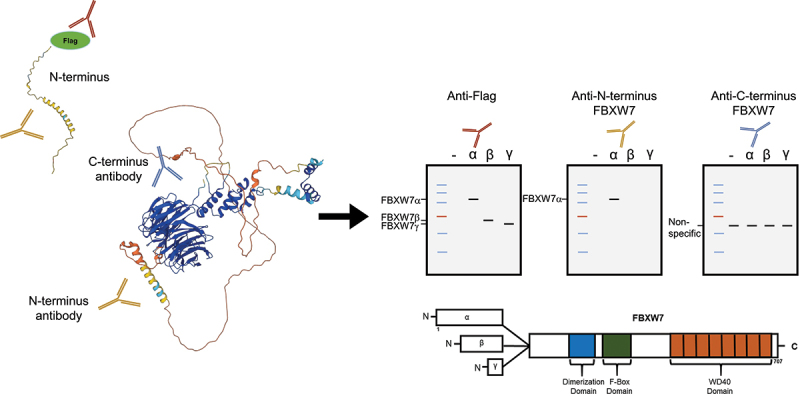
Predicted 3D tertiary structure of the N and C terminus regions of FBXW7α. Schematic comparing the tertiary structure of the *N*- and C- terminus regions of FBXW7α. The N-terminus sequence of FBXW7α, predicted by Alpha Fold AI, is a long linear and disorganized loop whereas the C-terminus, which is common to all three isoforms, is embedded in a series of β-sheet conformations. The cartoon on the right shows the ineffectiveness of a C-terminus directed antibody to detect any of the FBXW7 isoforms. In contrast, an antibody directed against the N-terminus of FBXW7α only detects the α isoform.

Finally, in terms of why the FBXW7α N-terminus directed antibody is more efficient, it is notable that Alpha Fold structure prediction software of the protein’s 3D structure shows that the FBXW7α specific N terminal domain forms a long, linear and disorganized loop (Figure 6) [16]. This suggests that the structure of this domain would be very similar to the linear amino acid structure and epitope used to raise the antibody (amino acid residues 1–50). The C-terminus, in contrast, is involved in a highly organized 3D tertiary structure consisting of a series of β-sheets [15,16]. This would suggest that the 3D structure of the protein is needed for epitope formation and antibody recognition. In addition, the H bonding maintaining this tertiary structure would be susceptible to heat-induced unfolding, perhaps preventing it from being detected after heat treatment as we describe here. Notably, since submission of this paper, the C-terminus antibody has now been discontinued due to its non-specificity and is no longer available (the producer AbCam notes it as being off catalog since November 2022, www.AbCam.com).

In summary, as a protein with a major function in cell growth and differentiation control, a better understanding of how FBXW7 is regulated will reveal new avenues for modifying its function in disease. Here we reviewed a commonly used antibody to FBXW7 for its effectiveness to detect FBXW7 in EC and found that it does not detect any of the three FBXW7 isoforms processed by conventional western blotting but rather detects a nonspecific band at 64kDa. Our findings show that published research reporting changes in FBXW7 protein expression using the C-terminal FBXW7 antibody used here should be reconsidered in light of our findings.

## Data Availability

The data used to conduct this study are available from the corresponding author upon reasonable request.

## References

[1] Strohmaier H, Spruck CH, Kaiser P, et al. Human F-box protein hCdc4 targets cyclin E for proteolysis and is mutated in a breast cancer cell line. Nature. 2001;413(6853):316–322. 11565034 10.1038/35095076

[2] Welcker M, Clurman BE. FBW7 ubiquitin ligase: a tumour suppressor at the crossroads of cell division, growth and differentiation. Nat Rev Cancer. 2008;8(2):83–93. 18094723 10.1038/nrc2290

[3] Welcker M, Larimore E, Swanger J, et al. Fbw7 dimerization determines the specificity and robustness of substrate degradation. Genes Develop. 2013;27(23):2531–2536. 24298052 10.1101/gad.229195.113 PMC3861666

[4] Grim JE, Gustafson MP, Hirata RK, et al. Isoform- and cell cycle–dependent substrate degradation by the Fbw7 ubiquitin ligase. J Cell Bio. 2008;181(6):913–920. 18559665 10.1083/jcb.200802076 PMC2426948

[5] Welcker M, Orian A, Grim JA, et al. A nucleolar isoform of the FBW7 ubiquitin ligase regulates c-Myc and cell size. Curr Biol. 2004;14(20):1852–1857. 15498494 10.1016/j.cub.2004.09.083

[6] Stephenson SEM, Costain G, Blok LER, et al. Germline variants in tumor suppressor FBXW7 lead to impaired ubiquitination and a neurodevelopmental syndrome. Am J Hum Genet. 2022;109(4):601–617. 35395208 10.1016/j.ajhg.2022.03.002 PMC9069070

[7] Popov N, Herold S, Llamazares M, et al. FBW7 and USP28 regulate myc protein stability in response to DNA damage. Cell Cycle. 2007;6(19):2327–2331. 17873522 10.4161/cc.6.19.4804

[8] Khan OM, Carvalho J, Spencer-Dene B, et al. The deubiquitinase USP9X regulates FBW7 stability and suppresses colorectal cancer. J Clin Invest. 2018;128(4):1326–1337. 29346117 10.1172/JCI97325 PMC5873885

[9] Tetzlaff MT, Yu W, Li M, et al. Defective cardiovascular development and elevated cyclin E and NOTCH proteins in mice lacking the FBW7 F-box protein. Proc Natl Acad Sci U S A. 2004;101(10):3338–3345. 14766969 10.1073/pnas.0307875101 PMC373463

[10] Izumi N, Helker C, Ehling M, et al. Fbxw7 controls angiogenesis by regulating endothelial NOTCH activity. PLoS ONE. 2012;7(7):e41116. 22848434 10.1371/journal.pone.0041116 PMC3407154

[11] Ramasamy SK, Kusumbe AP, Wang L, et al. Endothelial NOTCH activity promotes angiogenesis and osteogenesis in bone. Nature. 2014;507(7492):376–380. 24647000 10.1038/nature13146 PMC4943529

[12] Cassavaugh JM, Hale SA, Wellman TL, et al. Negative regulation of HIF-1α by an FBW7-mediated degradation pathway during hypoxia. J Cell Biochem. 2011;112(12):3882–3890. 21964756 10.1002/jcb.23321 PMC3202039

[13] Flügel D, Görlach A, Kietzmann T. GSK-3β regulates cell growth, migration, and angiogenesis via Fbw7 and USP28-dependent degradation of HIF-1α. Blood. 2012;119(5):1292–1301. 22144179 10.1182/blood-2011-08-375014 PMC3352078

[14] Wong Waihay J, Qiu B, Nakazawa Michael S, et al. MYC degradation under low O2 tension promotes survival by evading hypoxia-induced cell death. Mol Cell Biol. 2013;33(17):3494–3504. 23816886 10.1128/MCB.00853-12 PMC3753854

[15] Hao B, Oehlmann S, Sowa ME, et al. Structure of a FBW7-SKP1-cyclin Ecomplex: multisite-phosphorylated substrate recognition by SCF ubiquitin ligases. Molecular Cell. 2007;26(1):131–143. 17434132 10.1016/j.molcel.2007.02.022

[16] Jumper J, Evans R, Pritzel A, et al. Highly accurate protein structure prediction with AlphaFold. Nature. 2021;596(7873):583–589. 34265844 10.1038/s41586-021-03819-2 PMC8371605

[17] Matesanz N, Park G, McAllister H, et al. Docosahexaenoic acid improves the nitroso-redox balance and reduces VEGF-Mediated angiogenic signaling in microvascular endothelial cells. Invest Ophthalmol Vis Sci. 2010;51(12):6815–6825. 20702831 10.1167/iovs.10-5339 PMC3055780

